# Supplementary material for: A fibrin enhanced thrombosis model for medical devices operating at low shear regimes or large surface areas
Source: PLoS Comput Biol. 2022 Oct 3;18(10):e1010277. doi: 10.1371/journal.pcbi.1010277 (PMC9560616; doi:10.1371/journal.pcbi.1010277)

**S1 Text. Mesh convergence analysis.**

To evaluate the influence of mesh resolution, we compared results using a coarser and finer meshes. The corresponding concentration fields of Factor IIa and Factor V were compared to reflect higher and lower diffusivities. Paraview was used to interpolate the cell values between meshes of different sizes. The comparison along the middle plane of the oxygenator is provided below.

The finer meshes captured more local features of the species; however, the general topology of the scalar fields was consistent. This comparison is very challenging as the simulation is fully reactive and the convergence may not be the same for each mesh size at the same point in time. In addition, the interpolation step that enables the comparison between meshes might introduce errors.

**Factor IIa Concentration at Mid Plane**

Baseline Mesh Density (Medium):


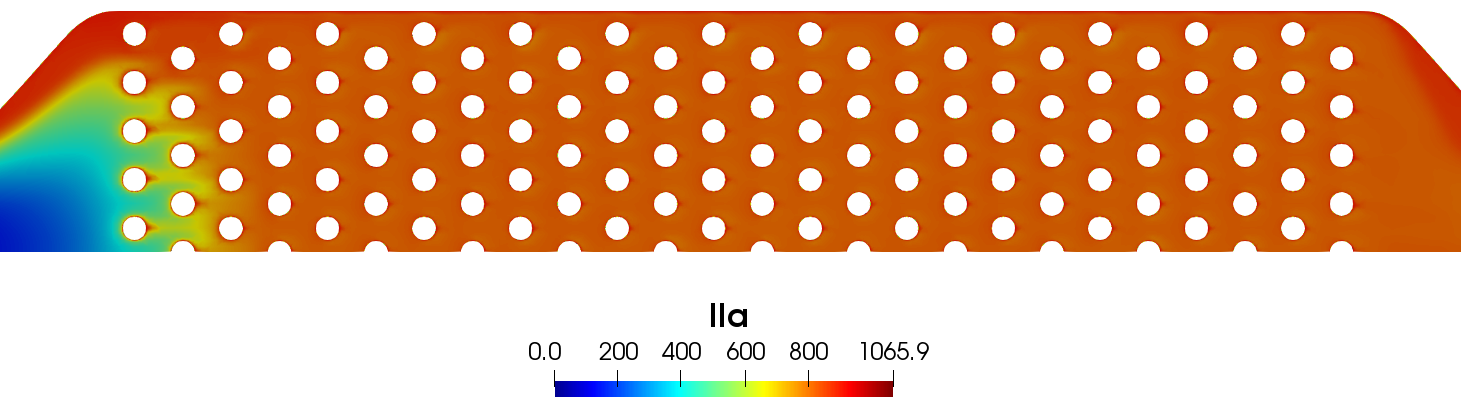


Coarse:


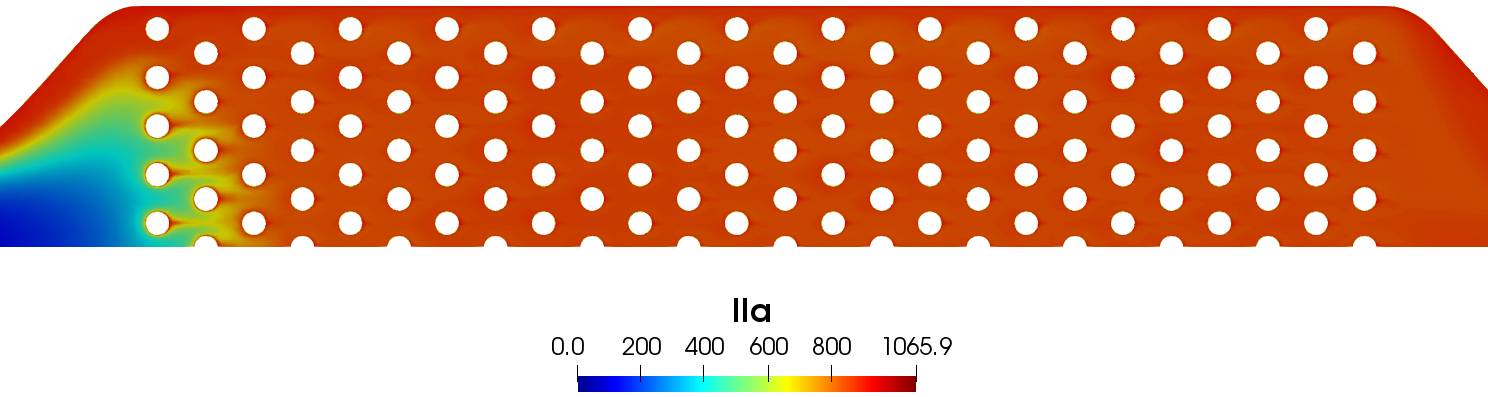


Fine:


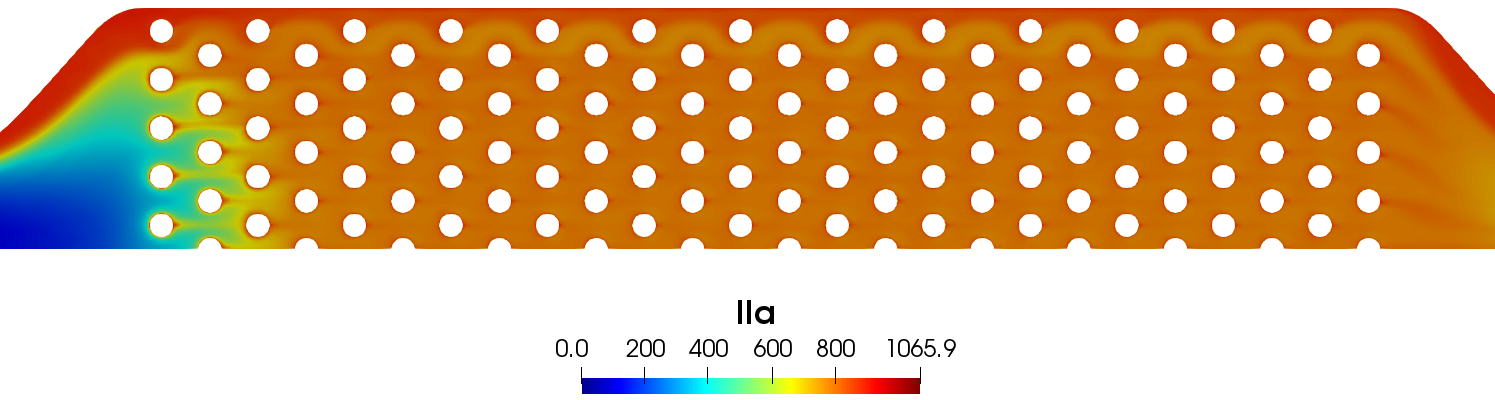


Difference Between Baseline and Coarse Mesh:


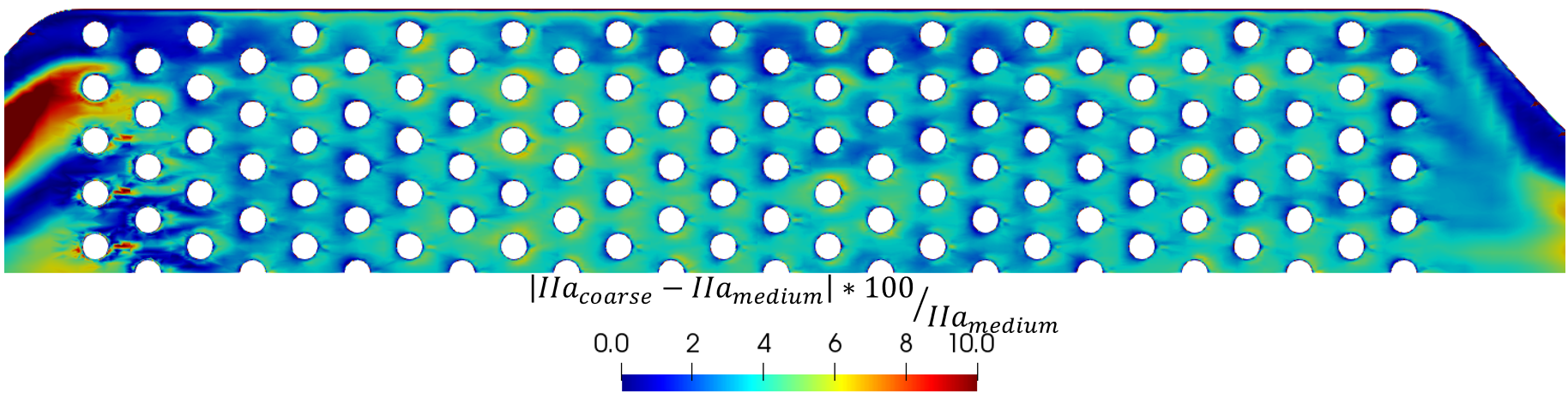


Difference Between Baseline and Fine Mesh:


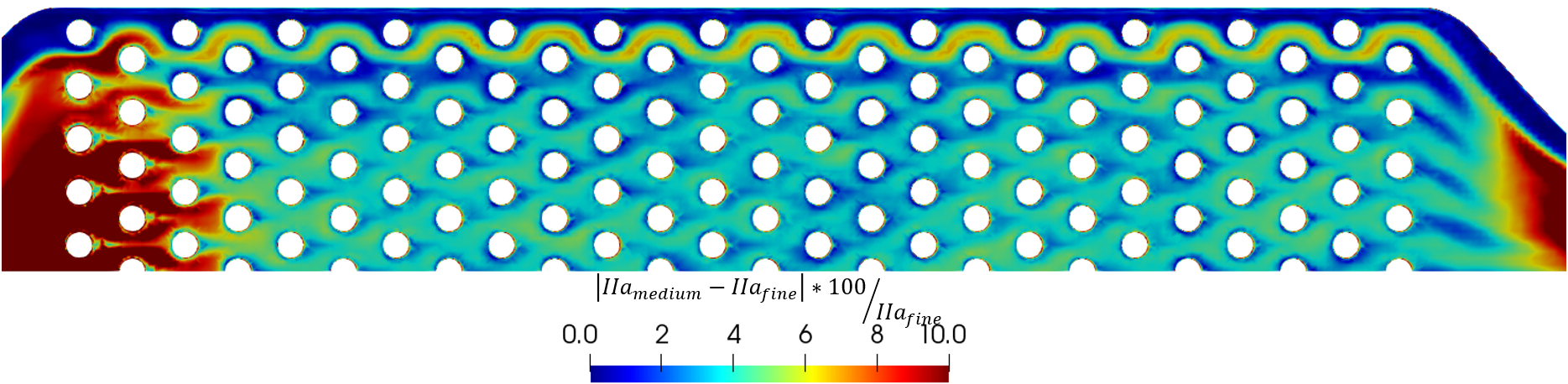


**Factor V Concentration at Mid Plane**

Baseline Mesh Density (Medium):


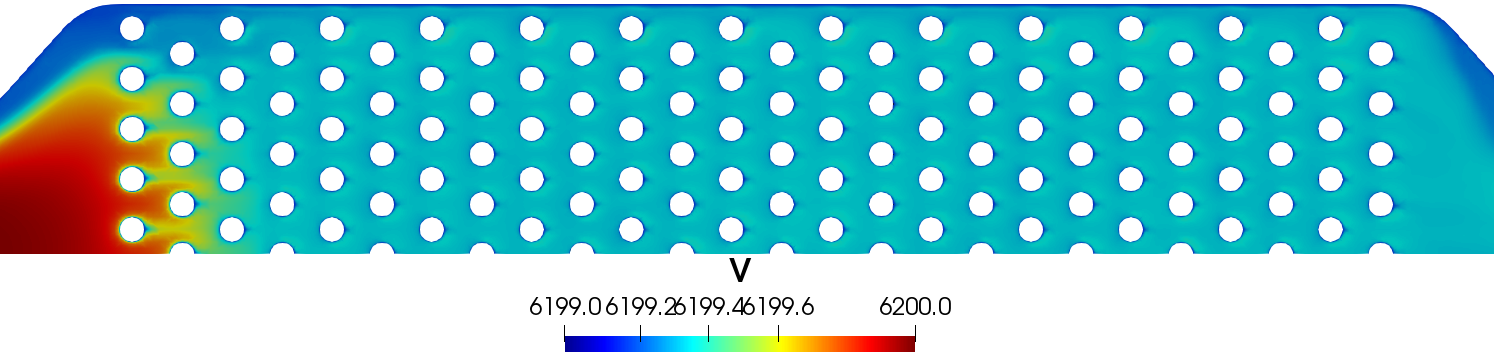


Coarse:


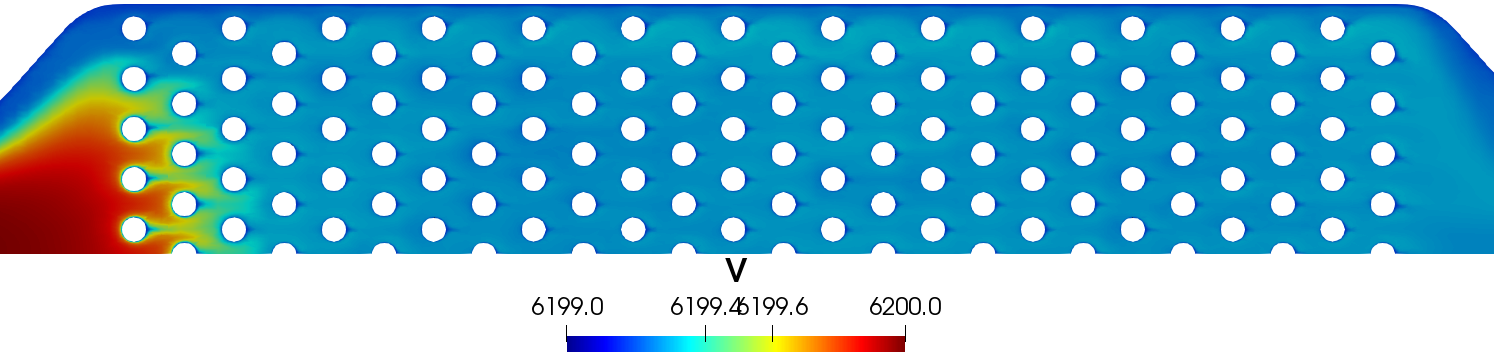


Fine:


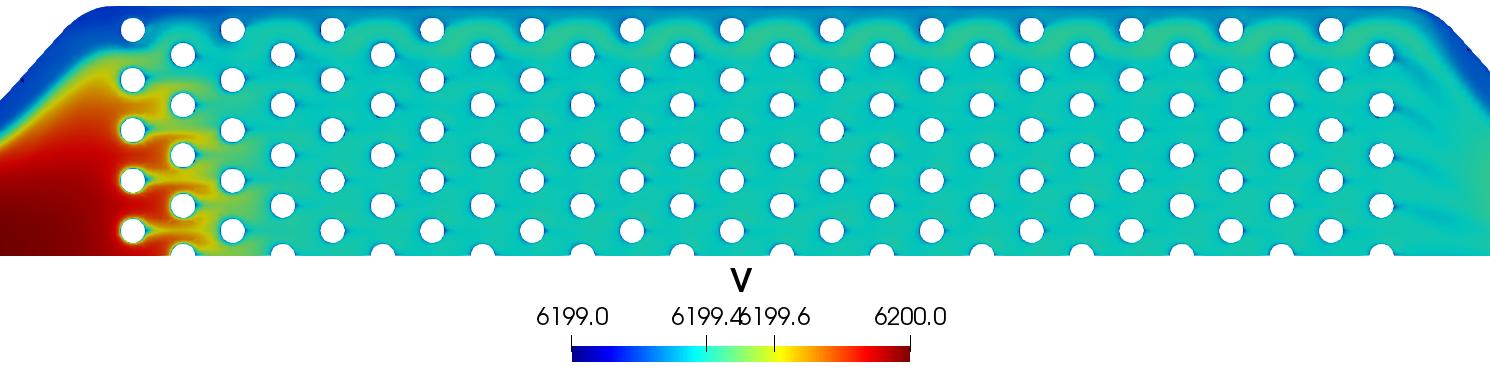


Difference Between Baseline and Coarse Mesh:


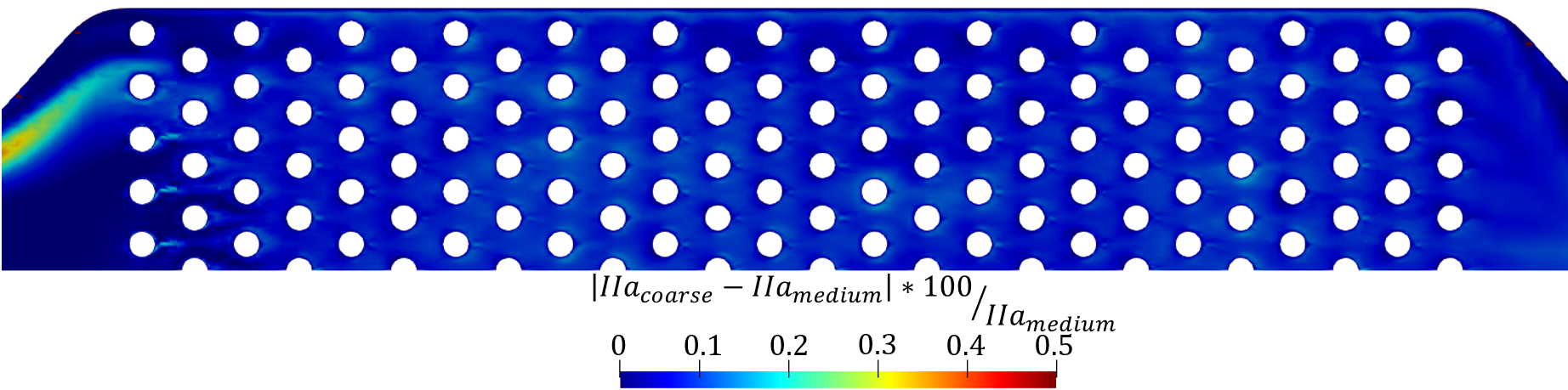


Difference Between Baseline and Fine Mesh:


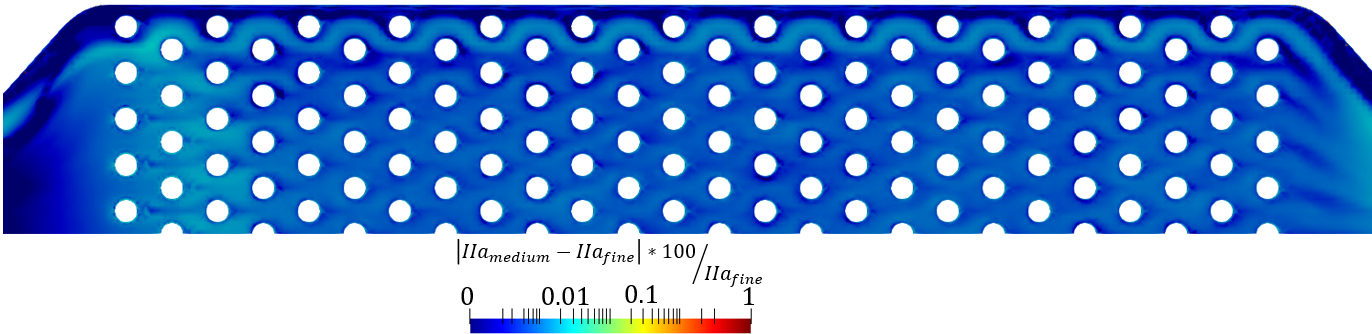

Supplement: S1 Text — A spatial grid analysis on the microfluidic fiber bundle using the concentration scalar fields of two coagulation factors is presented. (DOCX) [file pcbi.1010277.s001.docx]
