# Supplementary material for: A fibrin enhanced thrombosis model for medical devices operating at low shear regimes or large surface areas
Source: PLoS Comput Biol. 2022 Oct 3;18(10):e1010277. doi: 10.1371/journal.pcbi.1010277 (PMC9560616; doi:10.1371/journal.pcbi.1010277)
Supplement: S1 Fig — (PDF) [file pcbi.1010277.s002.pdf]

S1 Fig. Thrombus growth in Backward Facing Step reattachment point.

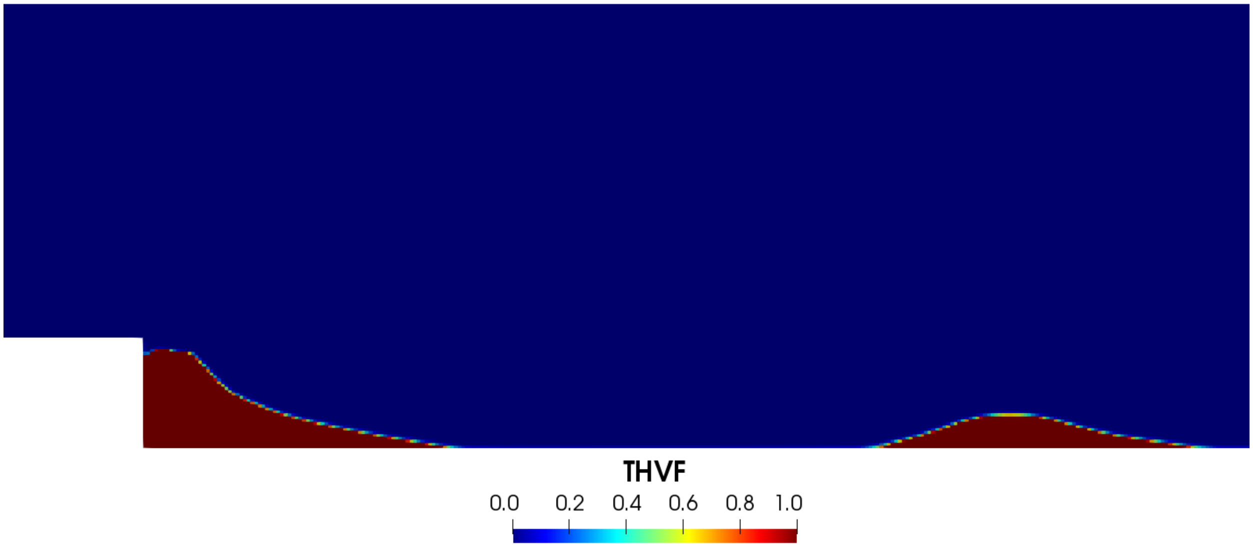

Thrombus Volume Fraction scalar field for human case at 1200 seconds.
