# Supplementary material for: A fibrin enhanced thrombosis model for medical devices operating at low shear regimes or large surface areas
Source: PLoS Comput Biol. 2022 Oct 3;18(10):e1010277. doi: 10.1371/journal.pcbi.1010277 (PMC9560616; doi:10.1371/journal.pcbi.1010277)
Supplement: Attachment — Submitted filename: Review1.docx [file pcbi.1010277.s003.docx]

We would like to thank the reviewers for their thoughtful critique of our submission, and for their helpful suggestions that have greatly improved the quality of the manuscript. Our responses are provided below in black, interposed with reviewers’ comments (red.) All changes to the main document are in red.

**Reviewer #1:**

This work by Méndez Rojano and colleagues presents a new thrombosis model developed to simulate clot formation in biomedical devices. To my knowledge, this is one of the most comprehensive thrombosis models currently available in the literature, as it includes platelet activation, platelet and fibrin deposition by low shear and through contact with reactive surfaces, also explicitly modelling (at least in part) the biochemical reactions of coagulation cascade, and allowing to predict thrombus composition. The model is then tested against the canonical backward facing step setup and against a hollow fiber membrane oxygenator, for which experimental results were available.

The authors effort to provide a comprehensive description and validation of the model is commendable. Despite failing to accurately capture some clot features observed in the experiments, the model itself is extremely promising and worth publishing at the current stage (the authors have addressed most of the limitations in the work discussion).

Major comments:

• Data and Code Availability: from PLOS CB code sharing Policy “PLOS Computational Biology requires authors to make all author-generated code directly related to their study’s findings publicly available without access restriction at the time of publication unless specific legal or ethical restrictions prohibit public sharing of code”. The authors shared their result files and post-processing codes. However, the thrombosis code is only available upon request, which constitutes an access restriction. Please make the thrombosis code and OpenFoam setup files freely available.

A github public repository containing the fibrin augmented thrombosis source code files is now included in the manuscript, along with a link to the openFoam V6 installation instructions – required to run the code. The *transportProperties* files including the thrombosis model parameters are included in the original data repository provided with the first version of this manuscript.

• Please double check consistency in variable naming and table numbers. This has made the work quite cumbersome to read. E.g., variables reported in Table 1 are not present (e.g., Spj, fstb) in Table 2 or might have changed names (e.g., krpd= krpdB?). Lines 156-160: Tables seem to be wrongly numbered. E.g. lines 157-158, the text refers to Table 6 for model parameters, but table 6 only reports inlet concentrations.

We thank the reviewer for these valuable observations that allow us to correct typos and missing elements in the original manuscript.

SpTxA2 was modified to Spj in the original manuscript to read Spj in both cases.

The variable fstb was originally present in the work of Wu et al. for platelet stabilization but was not considered in the current model, this mistake was corrected.

The definitions for krpd and kapd were added to Table 2. In addition, the table numbering in lines 157-158 was amended.

• Please provide more details about:

i) your modelling choices for reactive boundary conditions (Table 7); ii) cleaning by large shear for platelet and fibrin;

These two details have been added to the description of reactive boundary conditions in the backwards step subsection of the Methods. Briefly, the boundary conditions for the most part are adopted from Wu et al (2017) and supplemented with terms to account for the deposition factor XII and its conversion to XIIa, and likewise fibrinogen and fibrin. The cleaning by large shear rates for both platelets and fibrin mimics the effect of flow driven thrombus embolization and erosion, following the work of Goodman et al. (1) and Wu et al. (2).

[and] iii) the fibrin formation step (line 119)

The following sentence was added to the section *Coagulation cascade and fibrin/fibrinogen deposition.*

Fibrin formation was modeled through an enzymatic reaction taken from a simplified fibrin formation model developed by Chen et al. (3) conserving the Michaelis-Menten kinetic parameters.

• In the experiment, resistance across the device was measured, this can also be measured from the simulation results: how do the two compare?

Excellent question. We have added a paragraph describing the comparison of experimental to simulated occlusion. Briefly, the computational simulation significantly overpredicts the experimental results. We report this finding along with a possible explanation in the Discussion.

• Meshes: please report details regarding element dimensions in Table 8 and 10. Also, please specify the element type. As for the chosen parameters (recirculation length and pressure drop) for the mesh sensitivity tests, tests conducted using species concentrations (e.g., for the species with higher and lower D) would be more appropriate. I wonder if this might also be an additional reason why predictions are not perfectly in line with experimental results.

We have added the element type and dimensions.

We have also conducted two additional analysis using species concentrations for both higher and lower D (IIa and V) respectively at 1 s of thrombosis simulation. Factor IIa and Factor V concentrations were compared at middle plane. Paraview was used to interpolate the cell values between meshes of different sizes. These results are provided in new Supplementary Information section.

Although improving the mesh quality could lead to better experimental predictions, we believe that the main source of disagreement between simulations and experiments is related to the lack of some biophysical elements in the numerical model which are highlighted in the limitation section.

• Numerical setup - some details about the numerical setup are not reported: discretisation schemes, pressure-volume coupling scheme. Why was such a low time step (and CFL number) required to achieve numerical stability? Was the same time step approach used in both test cases?

The discretization and pressure-volume schemes were added in the manuscript. For time discretization and Euler scheme was used, for space discretization second order Gauss linear schemes were used. For scalar species bounded corrections were selected. The PIMPLE algorithm was used to solve the pressure-velocity coupling. An extremely low time step is not necessary, but it improves the stability of the simulation and requires less iterations to converge in the PIMPLE algorithm reducing the computational cost. A more detailed discussion on this dual time step strategy can be found in Mendez Rojano et al. (4). In both cases the same time step approach and discretization schemes were used.

Minor comments:

• Please report in the results or discussion the employed computational resources and simulation time. Thrombosis models tend to be quite computationally expensive and these information would help understand how close to actual R&D application the model is and to discuss future developments to make this possible.

This is an important point; accordingly, we added this information in the Methods. Briefly, we used a workstation PC with two Intel(R) Xeon(R) CPU E5-2699 v3 processors @ 2.30GHz with 18 cores and 36 threads. The 2D BFS thrombosis simulation took 3 hours wall time using 32 threads while the 3D fiber bundle chamber simulation took 126 hours wall time using 72 threads.

• Does the platelet to fibrin ratio influence thrombus resistance to flow?

Excellent question. In the current work we have adopted the original resistance term of Wu et al. (2) that considers the clot as a collection of sphere particles, we only modified the C_2_ constant value to represent a red clot in line with low shear thrombosis. We amended the limitations/future work section to acknowledge that the fibrin/platelet constituent ratio could be used to modify the resistance term towards a more comprehensive hinderance term.

• For future work, you might want to conduct a sensitivity test to better understand how choice of parameters influences your result. Could this also lead to a reduction in the number of equations?

This is an excellent suggestion; in fact we have conducted a global sensitivity analysis and an uncertainty quantification study on a previous version of our thrombosis model (4). In that study we found that a reduced set of parameters was influential to the quantities of interest suggesting that a reduced order model could be obtained. However, more work needs to be done to develop a proper reduced order model methodology for thrombosis models and achieve substantial reduction of computational times.

• Fig 2, caption: Fgd and Fnd are mentioned in the wrong order, I believe, creating some confusion.

We thank the reviewer for this remark, the error was corrected.

• Table 6 is mentioned in the text before Table 5

Tables were rearranged to amend the order.

• Micro-computed tomography: please specify how long the experiment lasted

We have added this information to the Methods. Briefly, the uCT scan itself took between 4 to 6 hours. The blood perfusion experiment took 15 minutes.

• Fig 6: no caption for part B

A caption was added to Fig 6 B.

• Could the authors discuss why it is important to get the thrombus time evolution right (and not just the end-result)?

This is a question we ask ourselves all the time. Does a patient really care if a stroke occurs at t=10s vs t=60s? On the other hand, the governing equations are all rate-based; therefore, it follows that the results should also be validated from the perspective of time/rate. Also, understanding why and at what time a device will clot can provide more information to improve designs and inform antiplatelet and antithrombin therapies.

On the other hand, we agree that it is critical to conduct the simulation (and experiment) to determine if the system reaches steady state, or if thrombus grows without bounds. (This is a major limitation of several prior thrombosis models.)

**Reviewer #2:**

Mendez Rojano et al. A fibrin enhanced thrombosis model for medical devices operating at low shear regimes or large surface areas

The manuscript continues the development of a computational thrombosis model with particular emphasis on fibrin deposition in low shear/large surface areas. The manuscript is well-written. The model is compared to existing published data from a backward facing step geometry and a microfluidic oxygenator. The model is based on a previous model and modified to be more sensitive to fibrin deposition.

Generally speaking, the model compares reasonably to the experimental data. Since the model is focused on the fibrin, only Figure 12 tests this aspect of the model in a rather qualitative manner. There is not a substantial amount of rigor associated with the validation but just one gross comparison that yielded similar results.

The Discussion section also does not really describe the fibrin-centric aspects with respect to the fibrin data presented. There is no quantitative analysis or comparison to demonstrate the overall accuracy of the model.

We thank reviewer #2 for the comments.

Although Figure 12 is the only figure that explicitly compares fibrin, one must consider that fibrin is embedded in the overall thrombosis model – hence critical to all key results presented here. For example, Figures 8 and 9 illustrates the importance of fibrin production to thrombus deposition, especially at low shear rates. This is a key contribution, as most existing models only consider platelet activity, hence will fail to predict the general thrombus growth trend. In platelet-based thrombus models even if the thrombus shape is correct, it would not be consistent with the composition of the thrombus.

We acknowledge that comparisons are based on gross patterns. In the discussion we qualify this fact by the difficulty of comparisons with fibrin or (any other biochemical species) scalar fields – hence their rarity in literature: both experiments and numerical simulations. To the extent of our knowledge, most mathematical thrombosis models only use the final form of the thrombus as a comparison. In this work we not only give both quantitative comparisons at different time points for the length and height of the thrombus in the backward facing step, but we also provide qualitative comparisons of the fibrin fields with fluorescent microscopy. Although we agree that a more rigorous validation is needed to advance the field, the results shown provide comparisons that do not exist in literature to the extent of our knowledge.

These above remarks have been incorporated into the discussion part of the manuscript.

**References**:

1.	Goodman PD, Barlow ET, Crapo PM, Mohammad SF, Solen KA. Computational model of device-induced thrombosis and thromboembolism. Ann Biomed Eng. 2005;33(6):780–97.

2.	Wu WT, Jamiolkowski MA, Wagner WR, Aubry N, Massoudi M, Antaki JF. Multi-Constituent Simulation of Thrombus Deposition. Sci Rep. 2017;

3.	Chen J, Diamond SL. Reduced model to predict thrombin and fibrin during thrombosis on collagen/tissue factor under venous flow: Roles of γ’-fibrin and factor XIa. PLoS Comput Biol. 2019;15(8):1–16.

4.	Méndez Rojano R, Zhussupbekov M, Antaki JF, Lucor D. Uncertainty quantification of a thrombosis model considering the clotting assay PFA-100®. Int j numer method biomed eng. 2022;38(5):e3595. Available from: https://onlinelibrary.wiley.com/doi/abs/10.1002/cnm.3595
